# Developing the First Telenursing Service for COVID-19 Patients: The Experience of South Korea

**DOI:** 10.3390/ijerph18136885

**Published:** 2021-06-26

**Authors:** Hyunsook Heo, Kyungyi Lee, Eunhee Jung, Hyangyuol Lee

**Affiliations:** 1 Comprehensive Community Care Center, Seoul National University Hospital, Seoul 03080, Korea; hsheo@snuh.org; 2 Nursing Service Department, Seoul National University Hospital, Seoul 03080, Korea; gyilee@snuh.org (K.L.); jungeh@snuh.org (E.J.); 3 College of Nursing, The Catholic University of Korea, Seoul 06591, Korea

**Keywords:** telehealth, telenursing, non-contact nursing counseling service, community treatment center, Republic of Korea, COVID-19

## Abstract

This study aimed to examine the process of establishing a telenursing service for COVID-19 patients with mild or no symptoms admitted to a community treatment center (CTC). The process of establishing the service was reviewed, and the degree of satisfaction with the provided service was investigated based on the medical records the patients submitted at their discharge from the CTC. A total of 113 patients were admitted; the patients themselves entered the self-measured vital signs and symptoms of COVID-19 infection to the electronic questionnaires and mobile application. The nurses implemented remote nursing based on the patients’ input data. The educational materials, including the video for self-measuring vital signs and the living guidelines, were prepared and arranged in advance. The telenursing protocol regarding the whole process from the patients’ admission to their discharge was used and applied to five other CTCs. The non-contact counseling service’s satisfaction and convenience scores were 4.65 points and 4.62 points, respectively, out of 5 points. The non-contact nursing counseling service played an important role in monitoring patients’ medical conditions during the spread of COVID-19. This experience of establishing telenursing services to the CTC provides a clear direction to innovate healthcare services in future disasters.

## 1. Introduction

As of 21 December 2020, the cumulative confirmed cases of COVID-19 in South Korea were 50,591, with 698 deaths and 35,155 quarantine-release cases [1]. After the first COVID-19 cases were confirmed in Wuhan, Hubei, China, in December 2019, the first case in Korea was confirmed on 20 January 2020, and the number of patients had drastically increased in the Daegu and Gyeongbuk regions by 18 February 2020. The infection transmission was so powerful and fast that all the patients diagnosed with COVID-19 were admitted to negative-pressure isolation units for treatment. However, due to the infection’s rapid transmission, the number of patients exceeded the available negative-pressure isolation units; thus, they began receiving treatment in normal beds as well. However, even normal beds were not sufficient in areas where mass infection occurred, and in some cases, patients waiting for hospital admission died in their homes during self-quarantine. The large-scale study conducted in China showed that 80% of confirmed COVID-19 patients were asymptomatic or only had mild symptoms, 14% of patients had severe symptoms, and the ratio of critical patients with a high fatality rate was about 5% [2]. On 1 March 2020, the Central Disaster and Safety Countermeasures Headquarters decided that patients with mild symptoms should be quarantined in facilities where they could receive care, and hospital treatments should apply only to severe and critical patients to reduce the number of COVID-19-related deaths, in response to the limited number of available hospital beds [3]. Therefore, the Community Treatment Centers (CTCs) began operating on 2 March 2020 to provide living and medical support to patients who had little demand for hospital treatments but required quarantine to prevent transmission and perform monitoring in state-operated facilities or other accommodation facilities.

The Seoul National University Hospital (SNUH) introduced a simultaneous operative system by opening a CTC for patients using the faculty-education facility in the Mungyeong Education Center, 170 km from the SNUH’s main campus, where the monitoring was performed. The Mungyeong CTC provided prompt counteractions related to patients’ medical conditions and other issues. In addition, diagnoses and tests were performed to investigate medical conditions while maintaining minimum contact with patients to prevent infection. The central monitoring headquarters in Seoul established a medical information system related to the non-contact nursing counseling service and non-contact doctor’s counseling service to monitor COVID-19 patients’ medical conditions in a non-contact manner. Medical formats for individual departments about patients’ conditions related to the infectious disease were developed for the non-contact nursing counseling service and guide brochures, videos, and examination protocols for patients were prepared.

Previous studies on nursing during a pandemic have been conducted on the psychological issues and effects based on patient care experiences [4,5], the demand for education about infectious diseases [6], and focus-group interviews about nursing experiences during a pandemic [7]. Most previous studies were conducted on nurses’ experiences after a pandemic or the psychological burden placed on nurses, but direct or non-contact nursing during a pandemic has not been studied.

The present study was conducted to investigate the process of establishing the non-contact nursing counseling service for COVID-19 patients with mild or no symptoms admitted to the CTC, which was first introduced in Korea, and to review the details and degree of satisfaction of the non-contact nursing service. This article provides the basic arrangements of the new nursing service for pandemic preparation and shares nursing experiences.

The present study includes the following specific objectives:

- To investigate the process of establishing the non-contact nursing counseling service;
- To review the contents of the educational materials and nursing service needed to establish the non-contact nursing counseling service;
- To analyze which factors associated with patients’ satisfaction with the non-contact nursing counseling service based on the discharge record sheets patients submitted at their discharge from the CTC.

## 2. Materials and Methods

### 2.1. Study Design

The present study is a retrospective study that used medical records to investigate the process of establishing the non-contact nursing counseling service for COVID-19 patients with mild or no symptoms that were admitted to the CTC, and to analyze which factors are associated with patients’ satisfaction with the non-contact nursing counseling service.

### 2.2. Subjects

The subjects of the present study included the process of establishing the non-contact nursing counseling service the CTC provided (operated by SNUH) and the 113 patients who were admitted to the Mungyeong CTC from 5 to 26 March 2020.

### 2.3. Non-Contact Nursing Counseling Service and Protocol

#### 2.3.1. Non-Contact Nursing Counseling Service

Monitoring the medical conditions of the COVID-19 patients with mild or no symptoms was performed by considering the three aspects described below. First, different roles were assigned to nurses and doctors. Second, the patients’ self-reported vital signs and information obtained through the non-contact counseling service were considered important. Finally, a protocol was prepared and applied to provide equal non-contact nursing counseling services to the patients, considering that the CTC nurses—who were not trained in advance but were volunteers from different workplaces—had to conduct the unfamiliar tasks.

**Figure 1:**
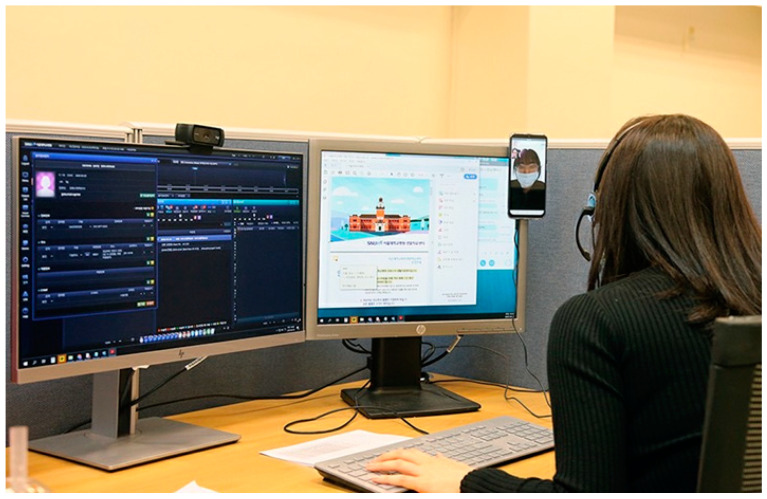
Providing a Telenursing Service for COVID-19 Patients.

The patients received the non-contact nursing counseling service once at the time of admission, twice a day periodically, and then whenever necessary (see Figure 1). Patients reported their blood pressure, temperature, heart rate, breathing rate, and oxygen saturation twice a day through self-measurement, which the nurses in charge reviewed. The vital signs guidelines for adults and children was prepared via collaboration with the relevant departments and nurses, with reference to the instructions from the Korea Disease Control and Prevention Agency. Nurses checked patients’ self-reported measurements through the non-contact nursing counseling service with reference to the vital signs guideline, and any re-measurement was requested when a measurement was out of the allowed range. A doctor would provide the non-contact counseling service if a patient’s re-measured vital sign was found to be out of the allowed range. A nurse obtained the patient’s medical information through the non-contact counseling service on the patient’s first admission day, including the patient’s vital signs and underlying diseases, which were considered important because the information served as a criterion to either conduct close-in monitoring for each patient during their stay in the CTC or to adjust their follow-up test schedule. Besides the periodic evaluations, vital signs were measured when a new symptom occurred, or an existing symptom became severe, and the information was passed to the doctor for examination. The non-contact nursing counseling service was provided a total of 7023 times. The non-contact doctor’s counseling service was provided a total of 1405 times, as it was performed on patients once at the time of their admission, once every two days periodically, and whenever necessary. Counseling related to the subjects’ daily living in the CTC, which was not related to their medical conditions, was provided a total of 950 times when the non-contact nursing counseling service was implemented.

The Mungyeong CTC, where the patients stayed, and the central monitoring center shared the information and modified the details on a real-time basis using a software program that all the CTC workers could access.

#### 2.3.2. Non-Contact Nursing Counseling Service Protocol

**Figure 2:**
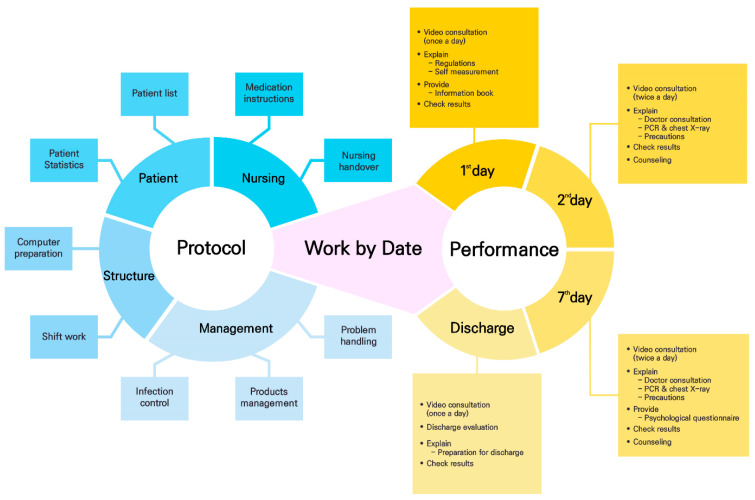
The protocol development process of Telenursing Service for the Community Treatment Center.

The nursing service protocol developed in the present study consisted of the following four domains: patient, nursing, structure, and management (see Figure 2). The patient domain comprised of a patient list, patient statistics, and medication information, and the nursing domain comprised of work by date, medication instructions, and nursing handover. The structure domain consisted of computer preparations by each nurse and shift work, and the management domain consisted of infection control, supply management, and problem handling. The work-by-date information included the non-contact nursing counseling service’s key activities.

- Day 1: The nurse in charge provided the video consultation (i.e., the initial evaluation) to the allocated patients to explain the living regulations and method of self-measurement; sent the video explaining the vital signs’ self-measurement method; checked the results on the basic admission information sheet, and provided information about the next day’s test.
- Day 2: A video consultation (i.e., the interim evaluation) was performed twice a day, and the information about the non-contact doctor’s counseling service and tests for individual patients was provided. In addition, counseling was performed regarding infection control, living in the CTC, and utilizing the facility.
- Day 7: In addition to the work performed on Day 2, information about a psychological test was provided.
- Discharge Day: A video consultation (i.e., the discharge evaluation) was performed, which offered details and guidance on preparing to leave the CTC.

A psychological questionnaire was sent to patients on their day of admission and then every 7 days to investigate the isolated patients’ psychological and emotional conditions. A patient was connected with a doctor when they complained about anxiety or psychological difficulties during the non-contact nursing counseling service. A psychiatrist conducted in-depth psychological counseling for high-risk patients. Patients received supplies from the National Center for Disaster Trauma as psychological and emotional supports, including URLs about daily exercise and meditation therapy.

### 2.4. Study Tools

Patients’ satisfaction with the non-contact examination was analyzed using two questions about the CTC and seven questions about the medical service the CTC provided based on the discharge record sheets patients submitted (see Table 1). The scores were calculated on a 5-point scale, with a higher score representing a higher degree of satisfaction.

### 2.5. Data Collection

The present study was conducted as a retrospective research by reviewing medical records so the subjects’ consent to participate in the study could be waived. However, the 113 subjects submitted their CTC entrance consents at their admission. The present study was conducted to investigate the process of establishing the non-contact nursing counseling service at the Mungyeong CTC from 5 to 26 March 2020. Data analysis was performed using the medical records (discharge record sheets) submitted by the 69 patients at their discharge from the CTC, excluding the questionnaires with redundant or missing responses.

### 2.6. Data Analysis

For data analysis, we used IBM SPSS Statistics for Windows (Version 25.0). First, descriptive statistics were conducted about the subjects’ ages and gender and the presence of a symptom in terms of frequency and percentage, and the degree of satisfaction with the non-contact examination was evaluated in terms of the mean and standard deviation. Second, Pearson’s correlation coefficients examined the relationships among the service factors related to the level of satisfaction and convenience of non-contact nursing service. Third, stepwise regression analyses were used to investigate factors associated with the non-contact nursing service’s overall satisfaction and convenience level (Q9 and Q10).

### 2.7. Ethical Considerations

The SNUH Institutional Review Board (IRB No. H-2106-155-1236) approved the present study.

## 3. Results

### 3.1. Establishing the Non-Contact Nursing Counseling Service System

A CTC examination department was generated to monitor the medical conditions of the patients admitted to the CTC. A new type of medical record and test code were generated to handle patients’ characteristics in relation to the infectious disease. The information system was established by considering that the patients should be able to understand infection-related information easily; the medical workers should be able to use the information and the information system should be interconnected with the hospital’s medical information system. Monitoring a patient’s medical conditions began when a patient transmitted the information about their self-measured vital signs and COVID-19 symptoms to the nurse. Counseling was provided via a medium that supported video and voice communication between a nurse and patient for non-contact monitoring based on the information the patients transmitted (Figure 1) [8]. In addition, nursing services were provided by partially utilizing a method for interconnecting the patient’s biological information with the hospital’s medical information system to record the data [9].

Because the non-contact nursing counseling service had to be established within a short period of 3 days, the format of transmitting patient data was changed in three stages (see Table 2). In the early stage, a questionnaire was prepared and used by employing a free survey software service based on the contents of newly prepared medical records. The questionnaire’s URL link was sent to the patients and their responses were manually recorded in the formats for each department. Next, non-contact nursing counseling was provided. In the middle stage, a newly developed electronic documentation was applied to interconnect patients’ input data with the hospital’s information system. In addition, a certification step to access the electronic documentation was provided to protect patients’ privacy, and a text message about the documentation input was automatically sent twice daily for the sake of work convenience. The nurses provided the non-contact nursing counseling with reference to the information interconnected with the hospital’s information system. The electronic documentation helped prevent manual input errors, reduce the medical workers’ workloads, and increase the convenience of the automatic data interconnection with the hospital’s computer network. In the final stage, an application based on a mobile phone platform was developed and used. The application included the functions for the infection information interconnected with the hospital’s medical information system, self-measured data input, information about the CTC, and one-to-one counseling and inquiry between the medical staff and patients.

In establishing the non-contact nursing counseling service at the central monitoring center, the number of necessary nurses required was calculated based on the 120 patients admitted to the CTC. The time required for the CTC entrance guidance, periodic evaluation, and discharge guidance for each patient was estimated to be 20 min; the periodic evaluation conducted twice a day and provisional evaluation were also considered. As a result, the nursing team comprised six day-shift nurses, six evening-shift nurses, and one charge nurse.

### 3.2. Formats for Individual Departments and Educational Materials for the Non-Contact Nursing Counseling Service

Monitoring the medical conditions of the COVID-19 patients with mild or no symptoms required minimal contact with them. Therefore, the necessary materials had to be prepared and arranged in advance. The doctors and nurses of the relevant departments prepared the newly developed medical record items while considering the symptoms of COVID-19, physical examinations, and mental health conditions. The items included the patients’ self-measured vital signs, major symptoms of COVID-19, underlying disease, medical history, physical examination, emotional state evaluation, and satisfaction with the CTC and non-contact examination. The following three medical records were generated at the patients’ admission: initial evaluation sheet (basic admission information sheet), interim evaluation sheet, and discharge evaluation sheet. Some items were modified for children, and they did not complete the discharge evaluation sheet. Print-out sheets were also arranged for patients who had difficulties using a smartphone, and temporary mobile phones were prepared for those without one.

A manometer, thermometer, and an oxygen-saturation meter were arranged in patients’ individual rooms for quarantine because monitoring their medical conditions relied on their self-measured vital signs. A video was prepared using the same devices arranged in the individual rooms for quarantine to explain the self-measurement method and how to use the devices. The video was sent to patients through their mobile phones upon their admission to the CTC. A brochure titled “Comprehensive Introduction to the SNUH CTC” was prepared and arranged in the individual rooms for quarantine to guide the patients from their admission to their discharge. The brochure included texts and images that provided details about the basic guidelines for quarantine, non-contact examination, methods for self-measuring vital signs, dining, fire-fighting measures, waste disposal, discharge from the CTC, and the CTC’s telephone numbers. The quarantine was implemented with one patient in each room at the CTC’s opening, but later, two patients had to share a room due to the increase in patients. Therefore, another brochure titled “Guideline for Sharing Rooms,” was prepared and distributed. In addition, the brochure titled “Instructions for Waste Disposal,” was prepared and provided to the patients regarding how to dispose of household waste and meal packages. The brochure titled “Guidelines for Discharge from the SNUH CTC” was prepared to explain how patients would be discharged from the CTC, with healthcare rules for patients to observe after their discharge and contact information of local health centers. All the materials and information distributed to the patients were prepared by referring to the instructions from the Korea Disease Control and Prevention Agency, counsels from the Infection Control Center, and opinions of local health centers.

### 3.3. Satisfaction and Convenience with Non-Contact Nursing Counseling Service

The discharge record sheets the COVID-19 patients submitted at their discharge were analyzed to investigate their satisfaction and convenience with the overall CTC quarantine life, non-contact examination, vital signs’ self-measurement method for monitoring symptoms, and the video guide detailing the self-measurement method. The overall CTC satisfaction and convenience scores were 4.45 points and 4.54 points, respectively, out of 5 points. The satisfaction and convenience scores for the non-contact counseling service via the video consultation system were 4.65 points and 4.62 points, respectively, out of 5 points. The satisfaction and convenience scores for the service, including the vital signs’ self-measurement method and medical staff’s monitoring abilities, were 4.52 points and 4.51 points, respectively, out of 5 points. The satisfaction and convenience scores for the video provided at admission about how to perform the vital signs’ self-measurement method were 4.71 points and 4.72 points, respectively, out of 5 points. The satisfaction score regarding the quality of the provided medical service was 4.62 points (see Table 3).

### 3.4. Patient Characteristics

A total of 113 patients diagnosed with COVID-19 were admitted to the CTC from 5 to 26 March [10]. The patients included 54 males (47.8%) and 59 females (52.2%). The average age of the patients was 30.4 years (from 9 to 65 years). The number of patients with a fever was one (0.9%) at admission, four within 3 days from admission (3.5%), and 15 within 2 weeks from admission (13.3%). No fever was found in 86.7% of patients. The symptoms frequently found in the patients included cough (27.4%), sputum (22.1%), and nasal discharge (15.9%). The patients’ underlying diseases included hypertension (n = 4, 3.5%), diabetes, asthma, and chronic bronchitis. The percentage of patients without a previously diagnosed disease was 93.8% (see Table 4).

### 3.5. Factors Associated with the Satisfaction and Convenience Level of Overall CTC Life

Table 5 shows the correlational coefficients among factors associated with patients’ satisfaction and convenience. The *r* coefficients for Q9 were significant in the range from 0.265 to 0.590 at the significant level of *p*-value 0.05. For Q10, the range was from 0.255 to 0.590 at the same level of significance.

### 3.6. Stepwise Regression for the Satisfaction and Convenience Level of Overall CTC Life

Table 6 and Table 7 reveal the regression results of the patients’ satisfaction and convenience levels about overall CTC quarantine life. The overall satisfaction level toward the video consultation system was the only significant predictor left in both models when explored with all different service factors (Q19 to 25). The factor increased by 0.583 points while 1 point of satisfaction increased in the satisfaction model, with a total explanation of 27.6% (*F* = 25.574; *p* < 0.01). In the convenience model, the same factor increased by 0.629 points when 1 point of convenience score increased, with a total explanation of 25.2% (*F* = 22.521; *p* < 0.01). This result means the successful video consultation system was the most significant and only factor for the satisfaction and convenience of overall quarantine life during patients’ admission at the CTC.

## 4. Discussion

The significance of the present study is that the first non-contact nursing service was applied in the Republic of Korea to patients with mild or no symptoms of COVID-19 who needed to be isolated and had limited access to healthcare services in a community setting. We verified that patients’ satisfaction and convenience levels resulted from the successful video consultation system.

As of 27 December 2020, the CTCs were located in 93 facilities throughout South Korea, with 11,720 rooms that could accommodate 17,749 individuals, and additional CTCs may open depending on the pandemic’s spread [11]. The CTCs were established to monitor the uncertain clinical courses of COVID-19 patients with mild or no symptoms [10] and efficiently distribute insufficient medical resources [3,9,10]. In addition, the CTCs have safely provided non-contact nursing counseling services to patients using information technology [9] and have protected healthcare workers from the pandemic.

Traditional non-contact nursing has reinforced nursing activities by adding a communication system to the areas of education, research, and management [8]. Non-contact nursing has been applied to nursing practices for the general population’s health management [12] and for patients with a cardiac disease [13] or mental health problem [14]. Since the COVID-19 pandemic, non-contact nursing has been introduced to elderly living facilities, nursing homes, or long-term management facilities to improve residents’ access to healthcare services [15] and has thereby been implemented extensively for subjects who live in an environment with limited access to healthcare services [16].

Telephone and video calls, websites, and other means of information technology have been used most frequently in non-contact nursing to collect patient information and transmit data to healthcare workers [8]. At the Mungyeong CTC, the non-contact nursing counseling service was provided by voice and video calls via smartphones between the patients and nurses. When necessary, text messages were also used to transmit and receive patient information for analysis and management. The non-contact counseling service system’s high satisfaction (4.65 points out of 5) and convenience (4.62 points out of 5) scores showed that patients had positive reactions to the non-contact counseling service, which allowed them to safely undergo examinations in isolation [17]. Considering that the average age of the patients was 30.4 years, the patients must have been familiar with the use of smartphones and were thus able to easily utilize the video consultation and nursing application. The high satisfaction (4.71 points out of 5) and convenience (4.72 points out of 5) scores for the video about how to self-measure vital signs suggest that videos may be effectively used in patient education or guidance. The results show non-contact nursing practices should include various methods to collect and transmit patient information according to the characteristics of individual patients [18] and to utilize cutting-edge information technologies [17].

In the CTC, nurses carried out essential front-line roles and communicated with patients firsthand. In situations where face-to-face communication was difficult, nurses employed means of discovering nonverbal patient information, besides the verbal information the patients transmitted via telephone, and managed the information effectively [19,20]. The necessary skills in non-contact nursing—beyond the work of connecting with the non-contact doctor’s counseling service—included effectively completing the patients’ evaluations, coordinating additional physical examinations, and special communication to carefully listen to the patients and ask appropriate questions [8,21]. The nurses working at the health promotion center volunteered to work at the CTC, and they satisfied the basic requirements for non-contact nursing, including three or more years of clinical experience, basic knowledge about nurse call-center procedures, and excellent customer service skills [21]. In non-contact nursing, situations may occur where a nurse is unable to directly verify or control the subjective information a patient provides, which can affect the patient’s final health outcome. Therefore, a nurse can ask effective questions about the patient’s subjective or objective information to differentiate which information is most critical to making a clinical decision about the patient’s treatment [22].

The preparation and application of the non-contact nursing protocol enabled the nurses to implement their tasks efficiently and apply consistent nursing service to the patients [19], helping the frequently replaced workers adjust to the tasks. The developed non-contact nursing protocol was successfully applied to four more CTCs the SNUH established. Non-contact healthcare, which may expand in the future, requires the development of the protocol and operational guideline, development, and education of specialized communication skills, and development of the detailed service model [9]. An ideal form of non-contact nursing should include advanced planning, detailed question structuring, consent preparation, a list for gathering necessary information, and a guideline for recording, which can be used as the basis of an insurance claim [15]. The American Nurses Association specified that the practice and clinical guidelines in the area of telenursing should be developed based on empirical evidence, when available, and on professional consensus among all involved healthcare disciplines, and that the guidelines’ developments should include cooperation with government agencies. In addition, the American Nurses Association also specified that nurses have the responsibility to develop an independent process to secure competencies in providing healthcare services [23]. The policies related to telehealth have been relaxed and extended in connection with the global COVID-19 pandemic [16]. After the pandemic, policies for telehealth management will need be established, and a discussion on the expense, results, and patient satisfaction is required for greater understanding [15]. Therefore, it is necessary to prepare a standardized course regarding the telenursing education curriculum, to establish a smart educational environment, and develop learning content through consultation with other disciplines [18]. Ideally, the state should establish a telenursing system that can manage citizens’ health conditions in normal situations and then adjust appropriately in disasters. To accomplish this goal, it is important to prepare protocols and guidelines for the organization and operation of nursing resources. Due to this current pandemic, it has emerged that telenursing can be expected to play a positive role in health coordination and general health management in the future.

This study has some limitations. First, this study was conducted retrospectively with institutional and governmental support but there was not enough time to establish the telenursing service’s process and to design the patient questionnaire for the study’s purpose. Second, this study sample size was too small to generalize the results. Third, this study was performed in the first CTC. A total of 17 CTCs now exist, which future studies can now examine.

## 5. Conclusions

The following suggestions derive from the results of the present study. First, a nursing protocol including telenursing services, as well as face-to-face nursing services, may be prepared and applied to efficiently implement a patient-centered individual approach in a disastrous situation outside of the normal healthcare environment or in cases where patients have difficulty visiting a hospital [9,18,24,25,26,27,28]. This approach is also important to acquire more accurate patient information at a reliable level. Second, the non-contact healthcare service was provided narrowly to COVID-19 patients only in the CTC, but despite legal problems, showed good results, including preventing the infection’s spread, the appropriate utilization of the limited supply of medical resources, and a high degree of patient satisfaction [17,18,19,22,25,28]. The definition of telemedicine in South Korea’s medical law is for medical personnel (medical doctors, dentists, and doctors of oriental medicine) to use information and communication technology to support medical knowledge or technology to people who require medical services in remote areas. There is no definition of any medical or nursing behaviors that patients may need [9,23]. The government has temporarily allowed patients to receive telephone counseling and prescriptions without visiting medical institutions, if safety is secured with the doctor’s discretion, in order to cope with the crisis of infectious diseases from COVID-19. Therefore, policies for appropriate and prompt healthcare interventions should be developed to respond to the rapidly changing healthcare environment.

## Figures and Tables

**Table 1:** The Discharge Survey questions.

Question No.	Contents
Q 9	Overall, how did you feel about the Community Treatment Center’s quarantine life? When you think that “you were very satisfied” with 5 points and “you are completely not satisfied” with 1 point, please indicate how satisfied you are.
Q 10	Overall, were you comfortable and was it convenient living in isolation at the Community Treatment Center? When you think “it was very convenient” with 5 points and “completely uncomfortable” with 1 point, please indicate the degree of convenience you felt.
Q 19	What did you think of the overall video consultation system? When you think that ‘you were very satisfied’ with 5 points and “you are completely not satisfied” with 1 point, please indicate how satisfied you are.
Q 20	Was the overall video consultation system convenient? When you think “it was very convenient” with 5 points and 1 point for “completely uncomfortable”, please respond to the level of convenience you felt.
Q 21	How did you feel about the self-monitoring service of vital signs (blood pressure, body temperature, etc.)? When you think that “you were very satisfied” with 5 points and “you are completely not satisfied” with 1 point, please indicate how satisfied you are.
Q 22	Was the self-vital-sign monitoring service convenient? When you think that 5 points for very convenient cases and 1 point for completely uncomfortable cases, please respond to the level of convenience you felt.
Q 23	What did you think of the video guide on how to measure vital signs at the time of admission? If you are very satisfied with 5 points and when you are not satisfied with 1 point, please answer the level of satisfaction you are satisfied with.
Q 24	Was the video guide on how to measure vital signs convenient? When you think “very convenient” with 5 points and 1 point for completely uncomfortable cases, please respond to the level of convenience you felt.
Q 25	How did you feel about the quality of the non-contact medical service via the video consultations you received? If you think that you are very satisfied with 5 points and when you are not satisfied with 1 point, please answer the level of satisfaction you are satisfied with.

**Table 2:** Changes in the medical records.

Characteristic	Early Stage	Middle Stage	Final Stage
Method	Record by hand	Electronic documentation	Application
Transmission	Send questionnaire URL	Automatically send URL	Push alarm
Record	Check results, Input, and sign	Check results and sign	Check results and sign

**Table 3:** Satisfaction in SNUH Community Treatment Center (SNUH-CTC) (*N* = 69).

Service Characteristic	Categories	Mean ± SD ^1^
Overall quarantine life in SNUH community treatment center	Satisfaction (Q9)	4.45 ± 0.65
	Convenience (Q10)	4.54 ± 0.74
Video consultation system	Satisfaction (Q19)	4.65 ± 0.59
	Convenience (Q20)	4.62 ± 0.62
Self-measured vital signs monitoring	Satisfaction (Q21)	4.52 ± 0.66
	Convenience (Q22)	4.51 ± 0.68
Vital signs measurement video guide	Satisfaction (Q23)	4.71 ± 0.64
	Convenience (Q24)	4.72 ± 0.64
Video consultation quality (Q25)		4.62 ± 0.67

^1^ SD = standard deviation.

**Table 4:** Patients’ general characteristics (*N* = 69).

Characteristic	Categories	*n* (%)
Sex	Male	54 (47.8)
	Female	59 (52.2)
Age(years) (Mean ± SD)		30.4 ± 12.9
Fever	At admission	1 (0.9)
	≤3 days	4 (3.5)
	≤2 weeks	15 (13.3)
	Never	98 (86.7)
Symptoms at admission	Cough	31 (27.4)
	Sputum	25 (22.1)
	Rhinorrhea	18 (15.9)
	Chest discomfort	8 (7.1)
	Sore throat	7 (6.2)
	Dyspnea	5 (4.4)
Underlying disease	Hypertension	4 (3.5)
	Diabetes	1 (0.9)
	Asthma	1 (0.9)
	Chronic bronchitis	1 (0.9)
	None	106 (93.8)

SD = standard deviation.

**Table 5:** Pearson’s correlation coefficients of the nine questions (*N* = 69).

	Q9	Q10	Q19	Q20	Q21	Q22	Q23	Q24	Q25
Q9	1	0.590 **	0.526 **	0.496 **	0.406 **	0.407 **	0.209	0.265 *	0.360 **
Q10	0.590 **	1	0.502 **	0.479 **	0.416 **	0.418 **	0.208	0.255 *	0.267 *
Q19	0.526 **	0.502 **	1	0.882 **	0.667 **	0.669 **	0.389 **	0.444 **	0.709 **
Q20	0.496 **	0.479 **	0.882 **	1	0.671 **	0.671 **	0.311 **	0.402 **	0.683 **
Q21	0.406 **	0.416 **	0.667 **	0.671 **	1	0.885 **	0.364 **	0.383 **	0.726 **
Q22	0.407 **	0.418 **	0.669 **	0.671 **	0.885 **	1	0.342 **	0.361 **	0.820 **
Q23	0.209	0.208	0.389 **	0.311 **	0.364 **	0.342 **	1	0.947 **	0.359 **
Q24	0.265 *	0.255 *	0.444 **	0.402 **	0.383 **	0.361 **	0.947 **	1	0.409 **
Q25	0.360 **	0.267 *	0.709 **	0.683 **	0.726 **	0.820 **	0.359 **	0.409 **	1

* *p*-value < 0.05, ** *p*-value < 0.01.

**Table 6:** A regression model of patients’ general satisfaction toward quarantine life in the CTC (Q9) (*N* = 69).

	B	SE	β	*t*	*p*
Constant	1.738	0.540		3.217	0.002
Q19	0.583	0.115	0.526	5.057	0.000
R^2^ = 0.276; Adj R^2^ = 0.265; *F* = 25.574 (*p* = 0.000).					

SE = standard error.

**Table 7:** A regression model of the convenience of quarantine life in the CTC (Q10) (*N* = 69).

	B	SE ^1^	β	*t*	*p*
Constant	1.612	0.621		2.594	0.012
Q19	0.629	0.132	0.502	4.746	0.000
R^2^ = 0.252; Adj R^2^ = 0.240; *F* = 22.521 (*p* = 0.000).					

^1^ Standard Error.

## Data Availability

The data are not publicly available due to privacy protections and restrictions from the institutional policy, which are supported by the Medical Law in South Korea.
